# Treatment of Ulcers by Circumcision

**Published:** 1873-11

**Authors:** E. T. Bruen


*MEDICAL TIMES:*

Treatment of Ulcers of the Leg by Circumcision.—*E. T. Bruen, M.D.*—In the issue of the *Medical Times* for June, 1873, an article appeared, taken from a German journal, stating that chronic, extensive, and otherwise intractible leg-ulcers had been successfully treated by an incision around the circumference of the ulcer down to the deep fascia. If care was taken to prevent union of the cut surfaces, a rapid cure was effected under the use of a simple water-dressing. Two cases were treated in the surgical wards of the Philadelphia Hospital during the past summer; and, thinking that the results might interest some, I shall give their history, as follows. Their perusal will probably indicate why further trial of circumcision was not made.

Charles Garton, æt. 61, admitted to the hospital January 21, 1873. He had a small ulcer situated on the anterior and outer surface of the leg, midway between the ankle and the knee. He was treated with various dressings, but without success, until last June, when it was determined to try circumcision. Under nourishing diet and suitable treatment, he gradually recovered his usual health; but before the spread of the ulcer was checked it had extended nine inches longitudinally, and five inches, or nearly around the limb, transversely. The surface has been slowly healing for the past eight weeks, and is now almost entirely well; but it occupied three months to restore him to the condition he was in before the trifling operation.

It does not appear to me that cell-change is sufficiently active, nor is the vitality of chronic leg-ulcers of such a character as to justify the danger of enlarging the pathological surface when the morbid conditions can be treated by numerous other measures not open to such objections. The subject is so trite that I should not have ventured to report these cases had I not been aware that the method of treatment has been somewhat discussed. It is proper to add that both these cases seemed favorable for a fair trial of this new mode of treatment, as both men enjoyed excellent health.

